# Vascularized Composite Allograft Rejection Is Delayed by Intrajejunal Treatment with Donor Splenocytes without Concomitant Immunosuppressants

**DOI:** 10.1155/2012/704063

**Published:** 2012-11-27

**Authors:** Christopher Glenn Wallace, Chia-Hung Yen, Hsiang-Chen Yang, Chun-Yen Lin, Ren-Chin Wu, Wei-Chao Huang, Jeng-Yee Lin, Fu-Chan Wei

**Affiliations:** ^1^Department of Plastic Surgery, Chang Gung Memorial Hospital, Chang Gung University and Medical College, 199 Tun Hwa North Road, Taipei 105, Taiwan; ^2^Department of Biological Science and Technology, National Pingtung University of Science and Technology 1 Hseuh-Fu Road, Neipu 91201, Pingtung, Taiwan; ^3^Department of Hepatogastroenterology, Chang Gung Memorial Hospital, Chang Gung University and Medical College, 199 Tun Hwa North Road, Taipei 105, Taiwan; ^4^Department of Pathology, Chang Gung Memorial Hospital, Chang Gung University and Medical College, 199 Tun Hwa North Road, Taipei 105, Taiwan; ^5^Department of Plastic Surgery, Chang Gung Memorial Hospital, Chia-Yi, Taiwan

## Abstract

*Background*. Mucosal or oral tolerance, an established method for inducing low-risk antigen-specific hyporesponsiveness, has not been investigated in vascularized composite allograft (VCA) research. We studied its effects on recipient immune responses and VCA rejection. *Methods*. Lewis rats (*n* = 12; TREATED) received seven daily intrajejunal treatments of 5 × 10^7^ splenocytes from semiallogeneic Lewis-Brown-Norway rats (LBN) or vehicle (*n* = 11; SHAM). Recipients' immune responses were assessed by mixed lymphocyte reaction (MLR) against donor antigen and controls. Other Lewis (*n* = 8; TREATED/VCA) received LBN hindlimb VCA and daily intrajejunal treatments of 5 × 10^7^ LBN splenocytes, or LBN VCA without treatment (*n* = 5; SHAM/VCA), until VCAs rejected. Recipients' immune responses were characterised and VCAs biopsied for histopathology. Immunosuppressants were not used. *Results*. LBN-specific hyporesponsiveness was induced only in treated Lewis recipients. Treatment significantly reduced MLR alloreactivity, significantly reduced VCA rejection on histopathology, and significantly delayed clinical VCA rejection (*P* < 0.0005; TREATED/VCA mean 9.6 versus 6.0 days for SHAM/VCA). Treatment significantly increased immunosuppressive IL-10/IL-4/TGF-**β** production and significantly decreased proinflammatory IFN-**γ**/TNF-**α**. *Conclusion*. Jejunal exposure to antigen conferred donor specific hyporesponsiveness that delayed VCA rejection. This method may offer a low-risk adjunctive treatment option to help protect VCAs from rejection.

## 1. Introduction 

The technical feasibility of transplanting vascularized composite allografts (VCA) such as of hand/forearm, larynx, partial face, and others is not disputed [1–5]. However, reconstructive VCA is unlikely to become widely available until either the risk profiles of lifelong immunosuppressant drugs become more acceptable or a safe method of donor-specific VCA tolerance induction applicable to humans is devised [6, 7]. Although transplantation tolerance has been established in many experimental models and anecdotal incidents of tolerance in humans can be found in the literature, efforts to replicate the state safely and reliably in humans have proven futile [8, 9].

Another method to reduce the attendant risks of nonspecific immunosuppression may be to induce donor-specific hyporesponsiveness [7, 10]. Although this is not transplantation tolerance, such a state may decrease the dosages required to maintain the allotransplant. Enteral (usually oral) administration of appropriate antigens can specifically suppress development or progression of experimental autoimmunities such as experimental autoimmune encephalitis, collagen-induced arthritis, nickel hypersensitivity and others [11, 12]. Human trials of oral tolerance have been conducted to treat allergies, rheumatoid arthritis, uveitis, diabetes, and other immunological diseases [11]. Others have shown that delivery of alloantigen to nongastrointestinal mucosa may be superiorly tolerogenic through avoidance of gastric acid and proteolytic enzymes [13–15]. Thus, Ishido et al. compared orally with intrajejunally administered donor splenocytes in a cardiac allotransplantation rat model and concluded from *in vitro *and *in vivo* evidence that jejunal mucosal tolerance was significantly more tolerogenic [16]. Importantly, their treatment protocol was commenced after transplantation and was donor specific since third party allotransplants were rejected normally [16, 17].

The effects of mucosal tolerance induction methods have not previously been assessed in a VCA model. Based on Ishido et al.'s investigations, we studied jejunal in preference to oral tolerance induction in a rat model of hindlimb VCA [16]. In contrast, to avoid counteraction with possible tolerization mechanism(s), we never administered immunosuppressive drugs; for this reason, we chose a semiallogeneic mismatch instead of a full mismatch. 

In this study, we aim firstly to confirm *in vitro* that intrajejunal treatments with donor splenocytes could render recipients immunologically hyporesponsive in a donor-specific manner. Second, we test this regimen *in vivo* to see if the commencement of rejection of semiallogeneic hindlimb VCAs could be significantly delayed. Third, further *in vitro* and *in vivo* analyses provide potential explanations for the underlying mechanisms of donor-specific hyporesponsiveness induced in recipients.

## 2. Materials and Methods

### 2.1. Animals

Adult (8–12 weeks old; 180–220 g) male inbred recipient Lewis (RT1^l^) and donor Lewis-Brown-Norway (LBN; RT1^l+*n*^) rats, representing a semiallogeneic mismatch, were obtained from the National Laboratory Animal Centre (Education Research Resource, Taiwan). They were housed individually in pyrogen-free conditions under controlled temperature and 12 hourly light/dark cycles, with water and commercial rat chow freely available at the Chang Gung Memorial Hospital Animal Centre. All experiments were authorised by and performed under instruction from the institution's Animal Care and Ethics Committee.

### 2.2. Study Design

**Figure 1:**
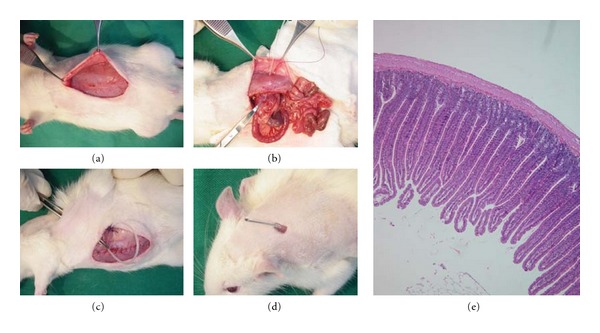
Percutaneous gastroduodenojejunostomy to deliver FDS or vehicle directly to the jejunal mucosa of Lewis rats. (a) Midline laparotomy. (b) Silicone tubing was delivered through the anterior abdominal musculature into the stomach and passed distal to the Treitz ligament. (c) The abdomen was closed and the tubing secured to abdominal wall musculature and tunneled to the posterior neck. (d) The tube was additionally secured at the posterior neck and sealed with a stopper that could be removed and replaced for intrajejunal administrations. (e) Characteristic jejunal histological appearance of intestinal tissue 5 mm distal to the tube end at time of sacrifice confirmed correct intrajejunal placement in all recipients.

Resealable percutaneous gastroduodenojejunostomies (Figure 1) were sited in all Lewis rats on Day −12 to establish direct access for intrajejunal administrations (Day 0 denoted the time of VCA or of animal sacrifice for one-way mixed lymphocyte reaction (MLR), depending on the Group to which the animal belonged). Two experimental (“TREATED” and “TREATED/VCA”) and two control (“SHAM” and “SHAM/VCA”) Groups were created as follows.

Lewis rats received 5 × 10^7^ LBN fresh donor splenocytes (FDS) in 0.2 mL HBSS intrajejunally (TREATED Group; *n* = 12), or vehicle alone (0.2 mL HBSS; SHAM Group; *n* = 11), everyday on Days −9 through −3 (7 doses) and were sacrificed on Day 0 for one-way MLR. This optimal dose of FDS for inducing hyporesponsiveness was characterized by preliminary studies that compared the effect of various doses (ranging between 1 × 10^7^ and 2 × 10^8^ FDS) administered for the same duration on Day 0 MLR responses versus vehicle-treated controls (data not shown).

Other Lewis rats received heterotopic LBN hindlimb VCAs on Day 0 and 5 × 10^7^ LBN FDS in 0.2 mL HBSS intrajejunally (TREATED/VCA Group; *n* = 8), or vehicle alone (0.2 mL HBSS; SHAM/VCA Group; *n* = 5), everyday from Day −9 until VCAs rejected. Importantly, immunosuppressive drugs were never administered.

### 2.3. Intrajejunal Access and Transplant Procedures

All operative procedures were performed aseptically with the animal deeply anaesthetized by intraperitoneal sodium pentobarbital (induction: 50 mg/kg; maintenance: 10 mg/kg/hr).

Resealable percutaneous gastroduodenojejunostomies were sited via standard midline laparotomies, securing the silicone tubing (model reference 806700; Shineteh Instruments Co. Ltd., Taiwan) palpably 1 cm distal to the ligament of Treitz. All tubes maintained this position as confirmed at animal sacrifice. The bowel 5 mm distal to the tube end was histologically confirmed in all sacrificed animals to be jejunum, confirming that treatment delivery was specifically to the jejunal mucosa and not more proximally (Figure 1).

**Figure 2:**
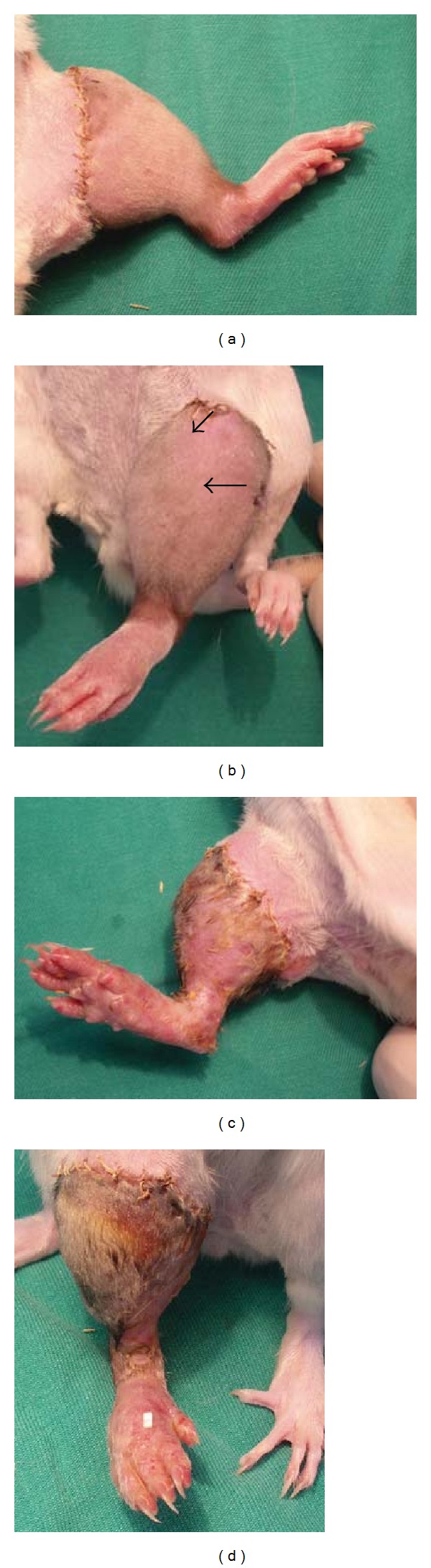
TREATED/VCA (panels (a) and (b)) and SHAM/VCA ((c) and (d)) on post-transplant Day +10. Arrows highlight the commencement of shedding of VCA hair in a TREATED/VCA rat. In contrast, recipients in the SHAM/VCA group ((c) and (d)) had commenced rejection at least three days previously and by now rejection was advanced.

Heterotopic hindlimb VCAs (Figure 2) were performed essentially as previously described [18]. All VCAs were revascularized after exactly 45 min of ischemia time by releasing both arterial and venous microvascular clamps at the designated time. All microanastomoses were complication-free and all VCAs maintained normal vascularity postoperatively. Donors and recipients were weight-matched to within 15 grams.

### 2.4. Preparation of Fresh Donor Splenocytes

Freshly harvested LBN whole spleens were gently mashed within serum-free RPMI-1640 and passed through nylon mesh (Millipore; 100 *μ*m pores) to produce single cell suspensions. Cells were washed with HBSS once and resuspended in ACK buffer for 5 min to lyse red blood cells. Cells were washed two times further with HBSS and resuspended at 5 × 10^7^ cells/0.2 mL HBSS. Splenocyte viability was >95% according to trypan blue dye exclusion.

### 2.5. One-Way Mixed Lymphocyte Reaction

MLR responses were assessed at the peak of the reaction as determined by preliminary study data (not shown); semiallogeneic MLR consistently provide counts that are not as high as fully allogeneic mismatched models.

One-way MLR was used to determine evidence of donor-specific hyporesponsiveness *in vitro*. On Day 0, spleens were freshly harvested for splenocytes from TREATED and SHAM Group recipients (responders), as well as naïve LBN (stimulator), and the animals sacrificed. 

Freshly harvested whole LBN spleens were gently mashed within serum-free RPMI-1640 and passed through nylon mesh (Millipore; 100 *μ*m pores) to produce single cell suspensions. Cells were washed with HBSS once and then resuspended in ACK buffer for 5 min. Cells were washed two times further with HBSS.

At this point, responder cells were resuspended in complete RPMI-1640 at 1 × 10^6^ cells/mL. Stimulator cells instead were treated with 5 *μ*m/mL mitomycin C in complete RPMI-1640 for 30 min at 37°C, washed twice with HBSS, and resuspended in complete RPMI-1640 at 1 × 10^6^ cells/mL.

Responder cells (1 × 10^5^ cells/100 *μ*L/well) were cultured in 96-well round-bottomed plates in triplicate with either: (1) equal numbers of mitomycin-C-treated stimulator cells (1 × 10^5^ cells/100 *μ*L/well; “SHAM + LBN Stim” and “TREATED + LBN Stim”); or (2) equal numbers of mitomycin-C-treated syngeneic cells (1 × 10^5^ cells/100 *μ*L/well; “TREATED Alone” and “SHAM Alone”); or (3) conclavulin A (ConA; 2 *μ*g/mL; “SHAM + ConA” and “TREATED + ConA”). Plates were maintained at 37°C in a 5% CO_2_ incubator for five days, consistent with the peak of the reaction according to preliminary studies. At 96 hr, cultures were pulsed with [^3^H]-thymidine (1 *μ*Ci/well) for 24 hr and harvested. Cell proliferation was assayed by (^3^H)-thymidine incorporation measured by *β*-scintillation counter. Two independent experiment repeats yielded essentially identical results.

### 2.6. Monitoring of Transplants and Recipients

Lewis rats were inspected daily for signs of graft-versus-host disease (GvHD): diarrhea, rash to the paws and/or ears, unkempt appearance, failure to thrive, and lack in weight gain. VCAs were monitored daily for markers of rejection (edema, erythema, desquamation, hair loss, epidermolysis, exudation, pustulation, and skin necrosis/escharification). Rejection was rigidly defined as “the first change in the skin after erythema and edema but before progressing toward epidermolysis, desquamation, or even eschar formation” [19]. The first specific sign was almost invariably the sudden shedding of hair specifically on the hindlimb VCA; this sign was both binary in its presence/absence and easily diagnosed with clarity. Rejection is defined by other authors as necrosis of 70–90% of the skin paddle of a VCA (which represents a nonsalvageable transplant), but we find this assessment to be more subjective in its interpretation than the presence/absence of hair shedding. Furthermore, in the clinical setting, acute rejection would be treated immediately rather than delayed in an attempt to reverse imminent VCA loss, and hence we believe the shedding of hair to be a more clinically relevant sign of rejection (and survival) than near-total necrosis.

### 2.7. Measurement of Cytokine Levels in MLR Supernatants and Day +7 Sera

Day +7 blood was obtained from each recipient's tail vein when VCA biopsies were performed. All sera and 96 hr MLR supernatants were stored at −80°C before assays, at which point they were gradually thawed. IFN-*γ*, TNF-*α*, IL-4, and IL-10 concentrations were measured by flow cytometric bead array (flow-CBA), whilst TGF-*β* concentrations were measured by enzyme-linked immunosorbent assay (ELISA). 

#### 2.7.1. Flow-CBA for Quantifying IFN-*γ*, TNF-*α*, IL-4, and IL-10 Concentrations

IFN-**γ**, TNF-*α*, IL-4, and IL-10 concentrations were quantified in multiplexed fashion in individual 96 hr MLR supernatant and Day +7 sera samples using the respective BD Biosciences CBA Flex Sets (Category Numbers: IFN-**γ**—558305; IL-10—558306; IL-4—558307; TNF-*α*—558309) according to the manufacturer's instructions. Briefly, 50 *μ*L of unknown samples, or standards, were added to premixed microbeads (50 *μ*L) in 12 × 75 mm Falcon tubes. After adding 50 *μ*L of a mixture of PE conjugated antibodies against the cytokines, the mixture was incubated for 3 hr in the dark at room temperature. This mixture was washed and centrifuged at 200 ×g for 5 min and the pellet resuspended in 300 *μ*L of wash buffer. The BD FACSCalibur flow cytometer was calibrated with setup beads and 1200 events were acquired for each sample. Individual cytokine concentrations were indicated by their fluorescent intensities (FL-2) and calculated using FCAP Array Software. Representative results from two independent experiments for sera and supernatants, respectively, which yielded identical results, were presented.

#### 2.7.2. ELISA for Quantifying TGF-*β* Concentrations

TGF-*β* concentration was quantified in 96 hr MLR supernatants and Day +7 sera by ELISA with specific antibody to TGF-*β* according to the manufacturer's protocol (Biosource International; Catalog no. KAC1688/KAC1689). For both cell culture media and sera, a sample extraction step was required to release TGF-*β* from latent complexes, making it accessible for measurement in the immunoassay. Representative results from at least two independent experiments (each performed in triplicate) for sera and supernatants, respectively, which essentially yielded identical results, were presented.

### 2.8. Histopathology of Rejecting Tissues

On Day +7, VCA-Muscle (9 mm^3^) and VCA-Skin biopsies (16 mm^2^) were obtained from the lateral aspect of the transplanted hindlimb and the wound closed (5/0 Vicryl; Ethicon). Biopsies were stored in 10% formalin for 36 hr, then embedded in paraffin, cross-sectioned, and stained with hematoxylin and eosin as standard. Lymphocyte counts per 0.1 mm^2^ field were assessed in quadruplicate per sample in muscle and at the dermal-subcutis interface in skin [20]. Histopathological analyses were performed, blinded by an independent pathologist. 

### 2.9. Statistical Methods

Data were expressed as mean ± standard deviation unless otherwise indicated. Statistical differences between groups were examined by Mann-Whitney *U*-Test, one-tailed (MWT-*ot*) or two-tailed (MWT-*tt*), and *t*-test as appropriate. Timing of VCA rejection was presented by survival curve using the product limit method of Kaplan-Meier and compared for differences using the logrank test. Statistical analyses were performed using SPSS software (Version 15). A statistically significant difference was indicated by a *P* value less than 0.05.

## 3. Results

### 3.1. Animal Monitoring

All animals remained entirely healthy throughout. In particular, no clinical evidence of GvHD was noted on daily evaluation and animals thrived and gained weight normally. All operations were complication-free.

### 3.2. Donor-Specific MLR Responses Reduced by Intrajejunal Administration of FDS

**Figure 3:**
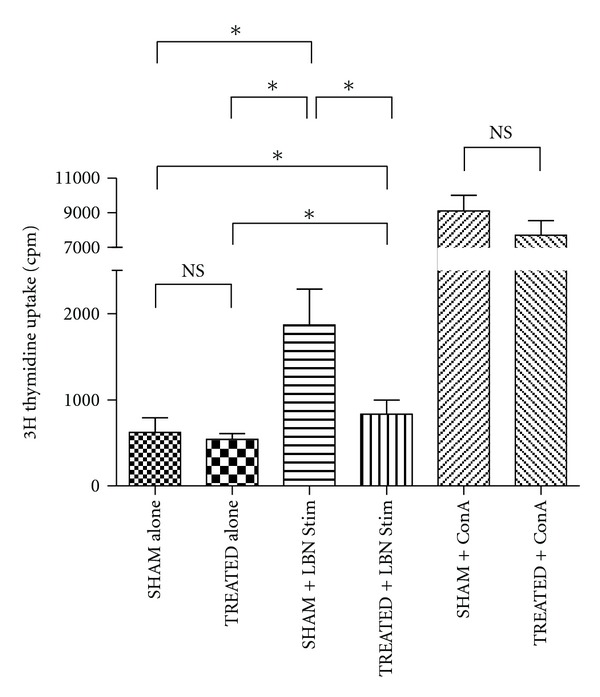
One-way MLR demonstrating significant donor-specific hyporesponsiveness conferred by seven consecutive daily intrajejunal doses of 5 × 10^7^ LBN FDS. “∗” *P* < 0.05; “NS” *P* < 0.05.

Splenocytes from TREATED rats showed suppressed MLR responses when used as responder cells with LBN as a specific stimulator (*P* < 0.05; MWT-*tt*; TREATED versus SHAM; Figure 3). Splenocytes from both SHAM and TREATED rats proliferated equally strongly against nonspecific ConA stimulation (*P* > 0.05; MWT-*tt*; SHAM + ConA versus TREATED + ConA). Splenocytes from SHAM and TREATED rats proliferated equally against syngeneic mitomycin-C-treated splenocytes (*P* > 0.05; MWT-*tt*; SHAM Alone versus TREATED Alone). 

### 3.3. Cytokine Profiles in MLR Supernatants

**Figure 4:**
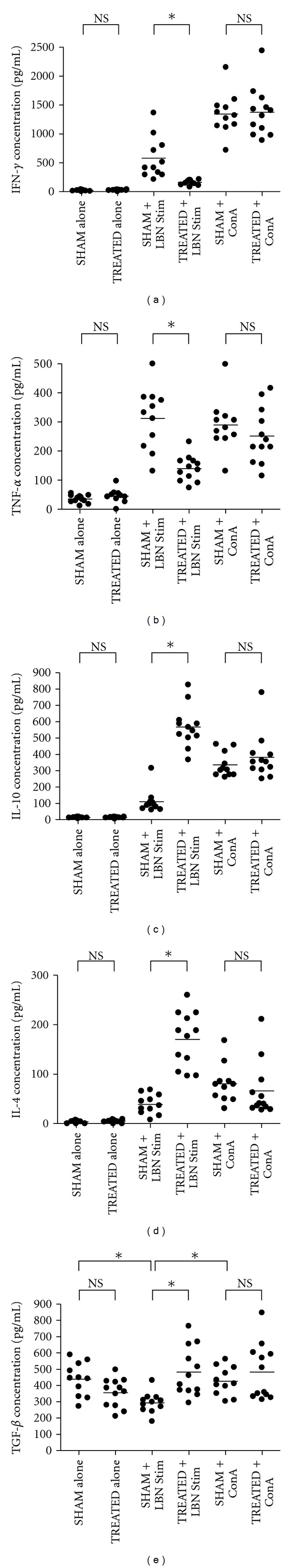
Cytokine concentrations in one-way MLR supernatants according to flow cytometric bead arrays ((a) IFN-*γ*; (b) TNF-*α*; (c) IL-10; (d) IL-4) and ELISA ((e) TGF-*β*) presented as scatter plots with mean bar. “∗” *P* < 0.05; “NS” *P* < 0.05 (specific *P* values provided in the text).

Concentrations of IFN-**γ** (*P* < 0.0001), TNF-*α* (*P* < 0.0001), and TGF-*β* (*P* < 0.001) in 96 hr supernatants were significantly reduced when splenocytes from TREATED rats were used as responder and LBN splenocytes as stimulator, whereas concentrations of IL-10 (*P* < 0.0001) and IL-4 (*P* < 0.0001) were significantly increased (each statistical comparison is TREATED versus SHAM using MWT-*tt*; Figure 4). When ConA was used as a non-specific stimulator of TREATED and SHAM splenocytes, 96 hr MLR supernatants did not reveal differences in IL-4, IL-10, TNF-*α*, TGF-*β*, or IFN-**γ** concentrations (*P* > 0.05; MWT-*tt*; TREATED Alone versus SHAM Alone). There were no differences in IL-4, IL-10, TNF-*α*, TGF-*β*, or IFN-**γ** concentrations when SHAM or TREATED splenocytes were cultured with syngeneic mitomycin-C-treated splenocytes (*P* > 0.05; MWT-*tt*; TREATED Alone versus SHAM Alone).

### 3.4. Onset of Rejection Delayed by Intrajejunal Administration of FDS

**Figure 5:**
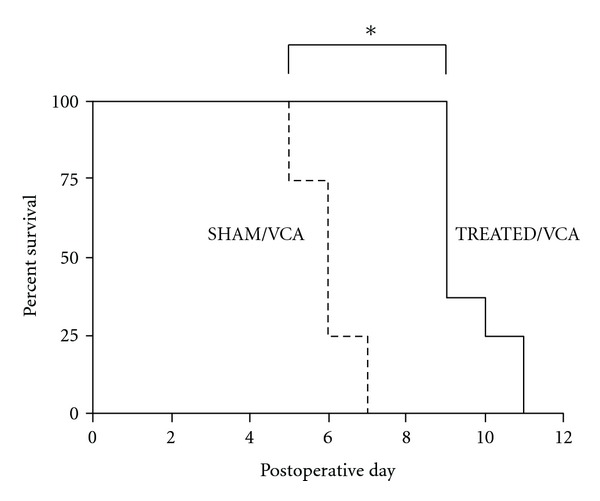
VCA rejection presented as survival curves using the product limit method of Kaplan and Meier. (dotted line—SHAM/VCA; solid line—TREATED/VCA; “∗” *P* < 0.0005).

Lewis VCA recipients were monitored daily for signs of rejection. When Lewis recipients were treated everyday by intrajejunal LBN FDS starting Day −9 before LBN hindlimb VCA (TREATED/VCA Group), VCA rejection was significantly delayed compared to untreated (SHAM/VCA) Lewis (*P* < 0.0005; mean 9.6 days versus mean 6.0 days; Figure 5).

### 3.5. Cytokine Profiles in Day +7 Sera of VCA Recipients

**Figure 6:**
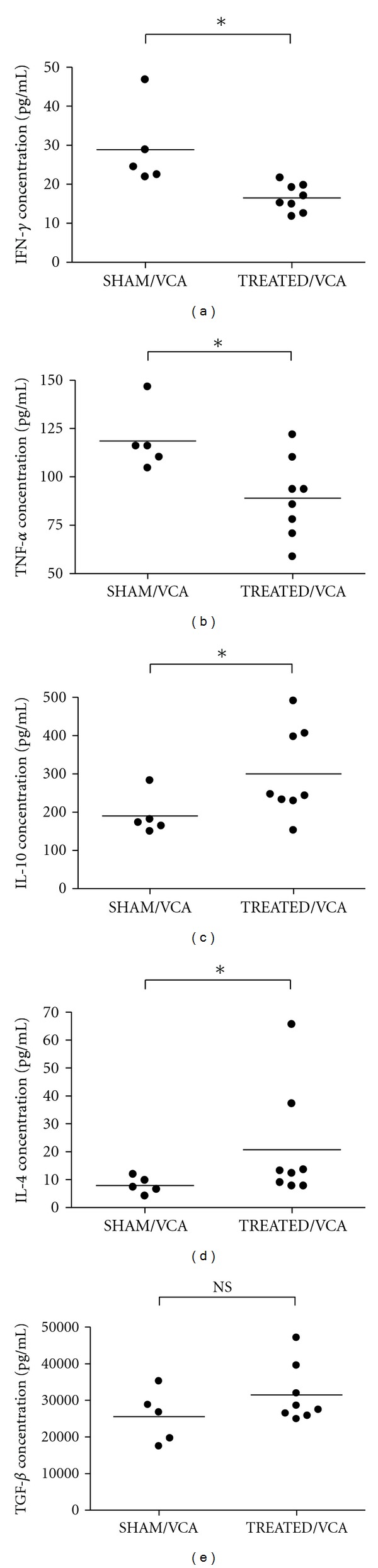
Cytokine concentrations according to flow-CBA ((a) IFN-*γ*; (b) TNF-*α*; (c) IL-10; (d) IL-4) and ELISA ((e) TGF-*β*) in Day +7 sera from TREATED/VCA and SHAM/VCA Groups presented as scatter plots with mean bar. “∗” *P* < 0.05; “NS” *P* < 0.05 (specific *P* values provided in the text).

When Lewis recipients were treated everyday by intrajejunal LBN FDS starting Day −9 before LBN hindlimb VCA, concentrations of IFN-**γ** (*P* < 0.005; MWT-*ot*; TREATED/VCA versus SHAM/VCA) and TNF-*α* (*P* < 0.05; MWT-*ot*; TREATED/VCA versus SHAM/VCA) in Day +7 sera were significantly reduced compared to Day +7 sera from untreated Lewis recipients, whereas concentrations of IL-10 (*P* < 0.05; MWT-*ot*; TREATED/VCA versus SHAM/VCA) and IL-4 (*P* < 0.05; MWT-*ot*; TREATED/VCA versus SHAM/VCA) were significantly increased. Differences in TGF-*β* concentration, however,did not reach statistical significance (*P* > 0.05; MWT-ot; TREATED/VCA versus SHAM/VCA; Figure 6).

### 3.6. Histopathology of VCA-Muscle and VCA-Skin

**Figure 7:**
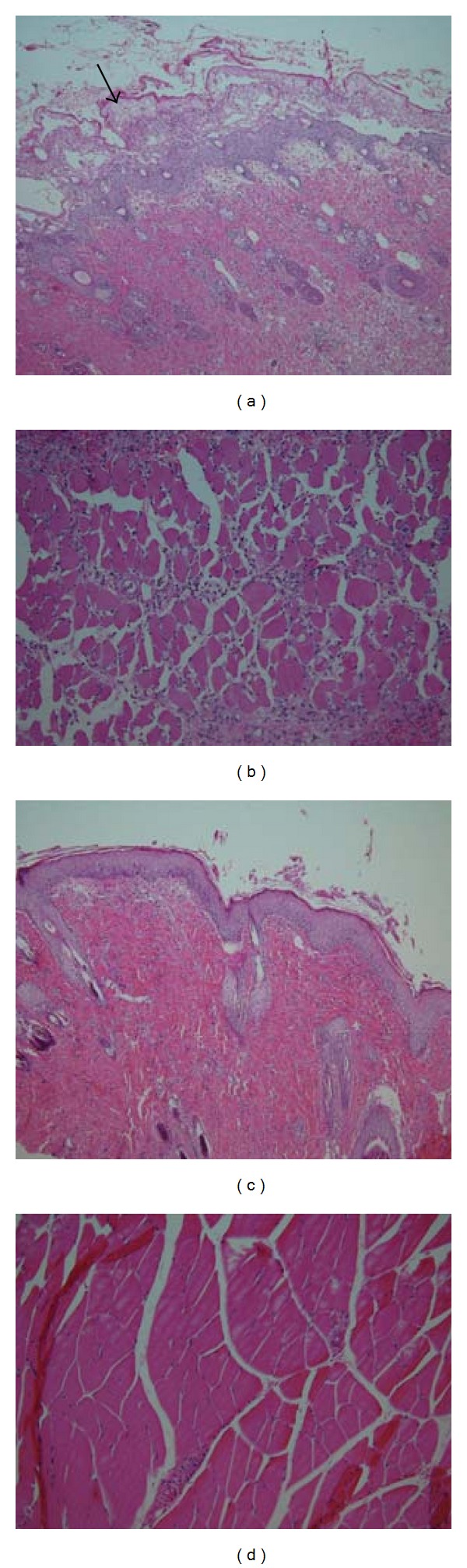
Photomicrographs (original mag: 200×) of Day +7 VCA-Skin ((a) and (c)) and VCA-Muscle ((b) and (d)) biopsies from SHAM/VCA ((a) and (b)) and TREATED/VCA ((c) and (d)) rats.

Biopsies from all recipients in TREATED/VCA and SHAM/VCA Groups were obtained from transplanted LBN hindlimbs on Day +7 and analyzed by an independent pathologist in blinded manner. VCA-Skin from SHAM/VCA all showed essentially the same characteristics: severe papillary edema with epidermal detachment, early necrotic changes in the superficial epidermis, and severe diffuse lymphocytic infiltration into all layers of the cutis and subcutis. VCA-Skin from TREATED/VCA rats, in contrast, revealed only mild papillary edema, essentially normal epidermal and dermal cytoarchitecture, properly adherent epidermis-dermis junction, and only mild focal lymphocytic infiltrates. Quantitatively, lymphocyte infiltration into the dermis-subdermis interface was significantly increased in SHAM/VCA compared with TREATED/VCA (*P* < 0.001; *t*-test; mean 60.6 ± 22.0  versus 23.9 ± 2.12 lymphocytes/0.1 mm^2^ resp.). VCA-Muscle from SHAM/VCA rats revealed generalized haphazard cytoarchitecture and severe diffuse lymphocytic infiltrate. VCA-Muscle from TREATED/VCA rats instead all revealed largely normal cytoarchitecture and only mild perivascular lymphocytic infiltrates. Quantitatively, lymphocyte infiltration into muscle was significantly increased in SHAM/VCA compared with TREATED/VCA (*P* < 0.001; *t*-test; mean 38.1 ± 11.7, versus 16.9 ± 1.8 lymphocytes/0.1 mm^2^ resp.). Representative samples are shown (Figure 7).

## 4. Discussion

This study demonstrated for the first time that daily intrajejunal administration of donor antigen could delay the rejection of hindlimb VCA despite the absence of immunosuppressant drugs. MLR demonstrated that recipient treatment with daily intrajejunal mucosal exposure to donor FDS suppressed alloimmune responses in a donor-specific manner. *In vivo*, this hyporesponsiveness manifested in delayed hindlimb VCA rejection as determined clinically and histopathologically. Cytokine concentrations in supernatants and recipients' sera showed decreased levels of proinflammatory IFN-*γ* and TNF-*α* and increased levels of immunosuppressive IL-10 and IL-4 in treated animals, but no differences in ConA non-specifically stimulated conditions in MLR, further suggesting the induction of a LBN-specific cytokine response. Although significantly elevated TGF-*β* levels in MLR supernatants suggested an immunosuppressive role for this cytokine in this model, this was not supported by* in vivo *data from sera. Further investigations into the role of TGF-*β* are warranted as cells predominantly secreting this cytokine may traffic extravascularly. Although this regimen did not produce tolerance, the hyporesponsiveness that was demonstrated was clinically relevant to VCA survival and was achieved purely by mucosal exposure to donor antigen.

Our findings support those of Ishido et al. who used a jejunal mucosal tolerization protocol in a cardiac allotransplantation model, although the immunogenicity of non-skin-bearing transplants (such as cardiac and renal) and skin-bearing VCA are known to be different [16, 21, 22]. Another critical difference between Ishido et al.'s and the present study was that the former used concurrent CsA therapy [16, 17]. Immunosuppressants such as CsA were not used in the present study because we hypothesized that nonspecific immunosuppression might interfere with the mechanistic establishment of hyporesponsiveness. Several mechanisms have been proposed to explain mucosal tolerance, including anergy, deletion of antigen-specific T cells, and induction of regulatory T cells (T-regs) [11]. Many different T-reg subtypes have been implicated in oral tolerance and in gastrointestinal immunoregulation, including: Th3, Tr1, CD4^+^CD25^+^ T, CD4^+^CD45Rb^low^, and CD4^+^LAP^+^ T cells [11, 23]. However, recent independent experiments in experimental and human transplant recipients have provided a strong evidence base that suggests calcineurin inhibition by CsA interferes with T-reg production, notably of CD4^+^CD25^+^FoxP3^High^ T-regs and the highly suppressive subset that are additionally CD27^+^ [24–29]. One such study suggested calcineurin-dependent IL-2 production was critically required for T-regs *in vivo*; the functional defect of T-regs after CsA exposure could be reversed by exogenous IL-2 [29]. Thus, CsA was omitted from our investigations in case antigen-specific T-reg production might also be important in this model and yet be abrogated by calcineurin inhibition. If our further investigations reveal a role for T-regs in this model, it will be important to determine whether they are spared by concurrent subtherapeutic rapamycin therapy instead of using CsA [24–29].

In the present study, it was demonstrated *in vitro *that intrajejunal LBN FDS caused upregulation of immunosuppressive cytokines IL-10, IL-4, and TGF-*β* with concomitant downregulation of proinflammatory IFN-*γ* and TNF-*α*. These findings were reflected in Day +7 sera from VCA recipients *in vivo*, except TGF-*β* levels at this time point were not significantly affected by treatment. These findings were largely in agreement with those of Ishido et al.; however they found no changes in TGF-*β* production in MLR supernatants, probably related to CsA administration [16, 27, 29]. Taken collectively, it seems likely that the present findings reflect the dominant involvement of donor-specific T helper-2 (Th2) cells although it is not yet possible to exclude secondary involvement of IL-10/TGF-*β* secreting T-regs in this system. In either circumstance, optimum dose, duration, and effective formulation of enteral antigen administration need to be determined to induce maximum antigen-specific immunosuppression and clarify the dominant mechanism [11, 30, 31].

Importantly, donor alloantigens can be delivered to the gastrointestinal mucosa in different forms, such as allogeneic cells or synthesized MHC proteins. Splenocytes from Lewis rats that had been fed donor splenocytes, their lysates, or synthesized donor MHC determinants, exhibited significant antigen-specific reduction of MLR responses *in vitro* and delayed-type hypersensitivity responses *in vivo* compared to unfed controls [19, 32–34]. Oral administration of a synthetic peptide (B7.75–84), corresponding to residues 75–84 of the human HLA-B7 molecule, to ACI rat recipients together with subtherapeutic cyclosporine A (CsA) caused Lewis cardiac allotransplants to survive indefinitely (>200 days) in 75% of recipients whilst third party skin allografts were rejected normally [35]. In a rat model of second-set rejection, oral administration of donor splenocytes prolonged semiallogeneic and fully allogeneic cardiac allotransplant survival times in an antigen-specific manner [19, 34]. Fully mismatched (BN to Lewis) renal allotransplant first-set survival times were significantly prolonged in recipients that had been prefed donor splenocytes and were prolonged further by donor cell feeds before and after transplantation [36, 37]. This prolongation was alloantigen specific and was accompanied by generation of intragraft CD8^+^ regulatory cells in tolerized animals [37, 38]. Adoptive transfer of these CD8^+^ cells to naïve rats transferred allotransplant tolerance observed in the original fed rats [38]. Other oral tolerization protocols have significantly prolonged survival times for nonvascularized allografts, including skin [39–44].

For a tolerance induction regimen to be applicable in humans, it must be safe. Intrajejunal access by endoscopically or fluoroscopically guided nasoenteric catheterization can be safely achieved in humans for up to 30 days [45]. Alternatively, donor antigens could be delivered in gastric-acid protected capsule form. Furthermore, no human trials of mucosal tolerance, which have tested a wide variety of antigens, have demonstrated toxicity from treatment or worsening of disease [11]. Since the dosage, type (e.g., whole cell or peptide; soluble or insoluble), and route of antigen delivery are critical to the mechanism and success of mucosal tolerance induction, variations in each of these and other parameters may be important in improving the tolerogenicity of mucosally delivered antigen whilst maintaining its safety [11, 30, 31].

It is conceivable, if further experiments confirm the safety, reliability, and underlying mechanisms of the approach, that early intrajejunal access and jejunal tolerance induction in human VCA recipients could be used to boost peripheral mechanisms of tolerance. Thereafter, although not yet investigated, it appears plausible that oral delivery of capsule-protected (against gastric acid) donor antigens could be used to maintain donor-specific hyporesponsiveness and allow reductions in immunosuppressive drug therapies. Additionally, antigen delivery as synthesized MHC peptides would likely be more acceptable to patients than enteral delivery of cell matter and requires further investigation.

## 5. Conclusion

The present study showed that intrajejunal administration of donor antigen induced donor-specific hyporesponsiveness with Th2 dominant status and delayed VCA rejection without concomitant immunosuppressants. Tolerance was not achieved but the hyporesponsiveness was clinically relevant and significant. Further investigations are warranted to optimize the administration (e.g., dose, form, and treatment duration) of alloantigens to maximize donor-specific hyporesponsiveness, changes to which may cause variations in the dominant mechanism(s) involved. Finally, the role, if any, and identification of various T-reg subtypes in this system invite clarification.

## Abbreviations

CBA: Cytometric bead array
ConA: Conclavulin A
CsA: Cyclosporine A
ELISA: Enzyme-linked immunosorbence assay
FDS: Fresh donor splenocytes
GvHD: Graft-versus-host disease
HBSS: Hanks balanced salt solution
IFN: Interferon
IL: Interleukin
LBN: Lewis-Brown-Norway
MHC: Major histocompatibility complex
MLR: Mixed lymphocyte reaction
MWT-*ot*: Mann-Whitney test one-tailed
MWT-*tt*: Mann-Whitney test two-tailed
TGF: Transforming growth factor
Th2: T helper-2
TNF: Tumor necrosis factor
T-reg: Regulatory T cell
VCA: Composite tissue allotransplantation.
